# Clinical study of skill assessment based on time sequential measurement changes

**DOI:** 10.1038/s41598-022-10502-7

**Published:** 2022-04-22

**Authors:** Tomoko Yamaguchi, Ryoichi Nakamura, Akihito Kuboki, Nobuyoshi Otori

**Affiliations:** 1 Institute of Advanced Biomedical Engineering and Science, Tokyo Women’s Medical University, 8-1 Kawada-cho, Shinjuku-ku, Tokyo, 162-8666 Japan; 2 Institute of Biomaterial and Bioengineering, Tokyo Medical and Dental University, Tokyo, Japan; 3 Department of Otorhinolaryngology, The Jikei University School of Medicine, Tokyo, Japan; 4 Graduate School of Science and Engineering, Chiba University, Chiba, Japan

**Keywords:** Biomedical engineering, Data processing, Health care

## Abstract

Endoscopic sinus surgery is a common procedure for chronic sinusitis; however, complications have been reported in some cases. Improving surgical outcomes requires an improvement in a surgeon’s skills. In this study, we used surgical workflow analysis to automatically extract “errors,” indicating whether there was a large difference in the comparative evaluation of procedures performed by experts and residents. First, we quantified surgical features using surgical log data, which contained surgical instrument information (e.g., tip position) and time stamp. Second, we created a surgical process model (SPM), which represents the temporal transition of the surgical features. Finally, we identified technical issues by creating an expert standard SPM and comparing it to the novice SPM. We verified the performance of our methods by using the clinical data of 39 patients. In total, 303 portions were detected as an error, and they were classified into six categories. Three risky operations were overlooked, and there were 11 overdetected errors. We noted that most errors detected by our method involved dangers. The implementation of our methods of automatic improvement points detection may be advantageous. Our methods may help reduce the time for reviewing and improving the surgical technique efficiently.

## Introduction

Endoscopic sinus surgery (ESS) is a common procedure for chronic sinusitis. In ESS, surgical navigation systems are often used, and their usefulness has been previously reported^1–3^. However, there are some reports of complications, such as diploma and cerebrospinal fluid (CSF) leak, although ESS utilizes a surgical navigation system^4,5^.

Improving the surgical outcome requires improvement of surgeons’ skills, although the rapid improvements in medical devices and a surgeon’s busy schedule make delivering effective training challenging. One of the training exercises for residents is “on the job training” (OJT), wherein the residents perform surgeries under the direction of experts. For OJT, the expert’s responsibility is to develop content for instruction, thereby creating significant challenges in the objectivity of the evaluation. For example, this factor can lead to variability in instruction and evaluation from person to person. In addition, this exercise adds extra burden on the experts.

Sugino et al.^6^ previously focused on positional information of surgical instruments during surgery and developed a time-series comparative analysis method. However, this method did not focus on detecting the erroneous aspects of the surgery. Aoki et al.^7^ developed a time-series comparative analysis method focused on motion data of surgical instruments and detected the time when the problem occurred. However, they analyzed only a limited number of scenes, instruments, and motion data.

Therefore, in our study, the time-series comparative analysis method was further developed into a multifaceted analysis focused on the motion and positional information of the surgical instruments. Furthermore, the effectiveness of our methods was verified by increasing the amount of data, compared to the amount used in previous studies.

We developed a system that could help perform a cross-sectional analysis of the ESS technique using the average value of the Surgical Feature Parameters (SFPs) in the selected surgical scene^8^. However, isolating the exact period of an error during a procedure using this system is difficult. Therefore, in this study, we aimed to develop a method that could help detect the exact period when the problem occurred.

## Methods

### Surgical information

We collected clinical data on image-guided ESS for cases of sinusitis in both nasal cavities, for which experts and residents treated the left and right nasal cavities. Experts suggested that the laterality of the measured cases, including difficulty, was negligibly small. Therefore, we focused on four basic scenes, as detailed in Table 1. Table 1 also shows the surgical instruments analyzed in each scene. The surgeries were performed at the Jikei University School of Medicine (Tokyo, Japan). Ethical approval was obtained from the ethics committee at the same institution [Approval Number 27-131(8016)].

**Table 1:** Definition of surgical scene and instruments used for measurements^6^.

Surgical scene	Task	Surgical instrument used for measurements	
		Right hand	Left hand
Scene 1*	Removal of nasal polyp	Microdebrider	Endoscope
Scene 2	Removal of the uncinate process		
Scene 3	Removal of the ethmoid bulla	Microdebrider and nasal cutting forceps	
Scene 4	Removal of third basal lamella		

*Only when a patient has nasal polyps.

### Tool motion acquisition

**Figure 1:**
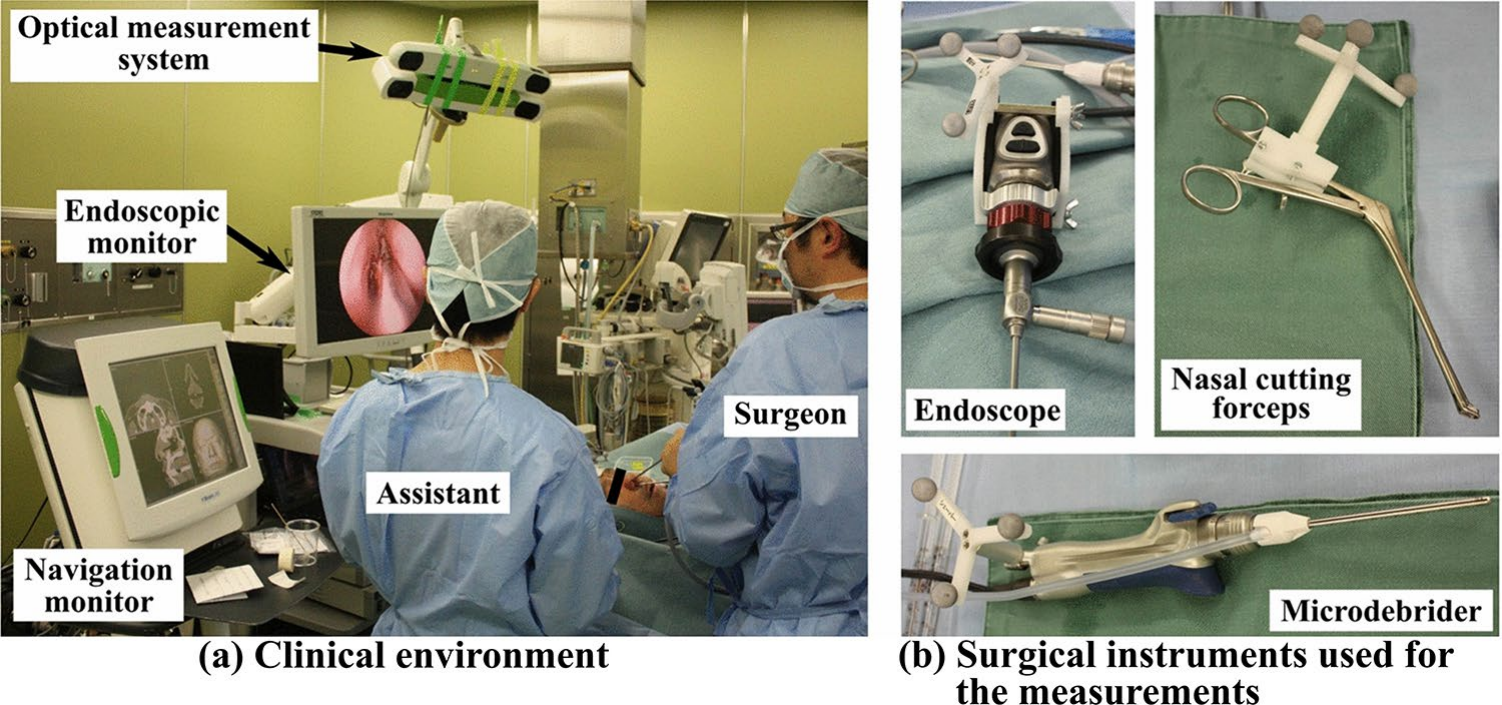
The setup for clinical study^7^.

Figure 1 shows the measurement setup. We used an optical position measuring device (Polaris Spectra System; Northern Digital, Inc., Waterloo, ON, Canada), which is also a commercially available surgical navigation system, to collect the surgery log data. The surgical log data consisted of the tip position of the surgical instrument P(= (x, y, z)), the rod orientation of the instrument $\vec{O}\left( { = \left( {ox,oy,oz} \right)} \right)$, the type of instrument, and the time stamp. We carried out point-based registration^9^ so that the tip position P was not in the coordinates in the real space, where the optical position measuring device was located, but in the coordinates in the patient’s computed tomography image space. Four feature points set on the patient’s face were used for this registration. In addition, we used reference markers to reduce errors because of a patient’s body movements. The sampling rate of the data used in this study was 5 Hz.

### Quantification of surgical features and creating the surgical process model

The surgical features, defined in a previous study^6^, are listed in Table 2. $P_{i} = \left( { = \left( {x_{i} ,y_{i} ,z_{i} } \right)} \right)$ is the position of the instrument tip in the *i*th frame $\left( {i = 1,2, \ldots ,n} \right)$. $L_{i} \left( { = P_{i} - P_{i - 1} } \right)$ is the distance from the instrument tip in the $\left( {i - 1} \right)$ frame to the distance at the $i$th frame. $\vec{O}_{i} \left( { = \left( {ox_{i} ,oy_{i} ,oz_{i} } \right)} \right)$ is the orientation vector of the instrument rod at the $i$th frame. $R_{{\text{i}}}$ in the rotation parameter denotes the degree of the angle of the instrument rod from the $\left( {i - 1} \right)$ frame to that in the $i$th frame. The parameters represented by *L* and *R* indicate the parameters of the instruments manipulated by the left and right hands, respectively. Aoki et al.^7^ only used velocity, rotation, relative velocity, and bimanual distance 1; however, we considered all parameters for the multilateral data analyses. We added acceleration and jerk, which are often used in surgical technique evaluation to evaluate finer parts of the movement of surgical instruments (e.g., smoothness of movement). In addition, in ESS, which handles lesions with a lot of bleeding due to inflammatory lesions in a narrow space in the nasal cavity, maintaining the sharpness of the camera is especially important for smooth surgical progress. Therefore, we considered it necessary to grasp the positioning of the instrument as well as the relative positional relationship among the instrument, lesion (i.e. nasal tissue), and the camera position as an element. Thus, the bimanual angle and bimanual distance 2 were added as indicators of the positioning of the two surgical instruments.

**Table 2:** Definition of surgical feature parameters^6^.

Operation features of the left or right hand		
Velocity (v [mm/s])	Rate of change in the position of the instrument tip	$v_{i} = \left| {\frac{{dL_{i} }}{dt}} \right|$
Acceleration (a [mm/s^2^])	Rate of change in the velocity of the instrument tip	$a_{i} = \left| {\frac{{dv_{i} }}{dt}} \right|$
Jerk (j [mm/s^3^])	Rate of change in the acceleration of the instrument tip	$j_{i} = \left| {\frac{{da_{i} }}{dt}} \right|$
Rotation (r [deg/s])	Rate of change in the orientation of the instrument rod	$r_{i} = \left| {\frac{{dR_{i} }}{dt}} \right|$ $\left( {R_{i} = \cos^{ - 1} \left( {\frac{{\overrightarrow {{O_{i} }} \cdot \overrightarrow {{O_{i - 1} }} }}{{\left| {\overrightarrow {{O_{i} }} } \right|\left| {\overrightarrow {{O_{i - 1} }} } \right|}}} \right)} \right)$
Bimanual operation feature		
Relative velocity (rv [mm/s])	Difference in the velocity between the endoscope and the instrument	$rv_{i} = v_{{R_{i} }} - v_{{L_{i} }}$
Relative acceleration (ra [mm/s^2^])	Difference in the acceleration between the endoscope and the instrument	$ra_{i} = a_{{R_{i} }} - a_{{L_{i} }}$
Relative jerk (rj [mm/s^3^])	Difference in jerk between the endoscope and the instrument	$rj_{i} = j_{{R_{i} }} - j_{{L_{i} }}$
Bimanual angle (ba [deg])	Angle between the endoscope rod and the instrument rod	$ba_{i} = \cos^{ - 1} \left( {\frac{{\overrightarrow {{O_{{R_{i} }} }} \cdot \overrightarrow {{O_{{L_{i} }} }} }}{{\left| {\overrightarrow {{O_{{R_{i} }} }} } \right|\left| {\overrightarrow {{O_{{L_{i} }} }} } \right|}}} \right)$
Bimanual distance 1 (bd1 [mm])	Distance between the endoscope tip and the instrument tip	$bd1_{i} = \left| {P_{{R_{i} }} - P_{{L_{i} }} } \right|$
Bimanual distance 2 (bd2 [mm])	Difference between the instrument tip and centerline, which passes through the center of the endoscope lens, parallel to the endoscope rod	$bd2_{i} = \frac{{\left| {\left( {\overrightarrow {{P_{{R_{i} }} - P_{{L_{i} }} }} } \right) \times \overrightarrow {{O_{{l_{i} }} }} } \right|}}{{\left| {\overrightarrow {{O_{{L_{i} }} }} } \right|}}$

**Figure 2:**
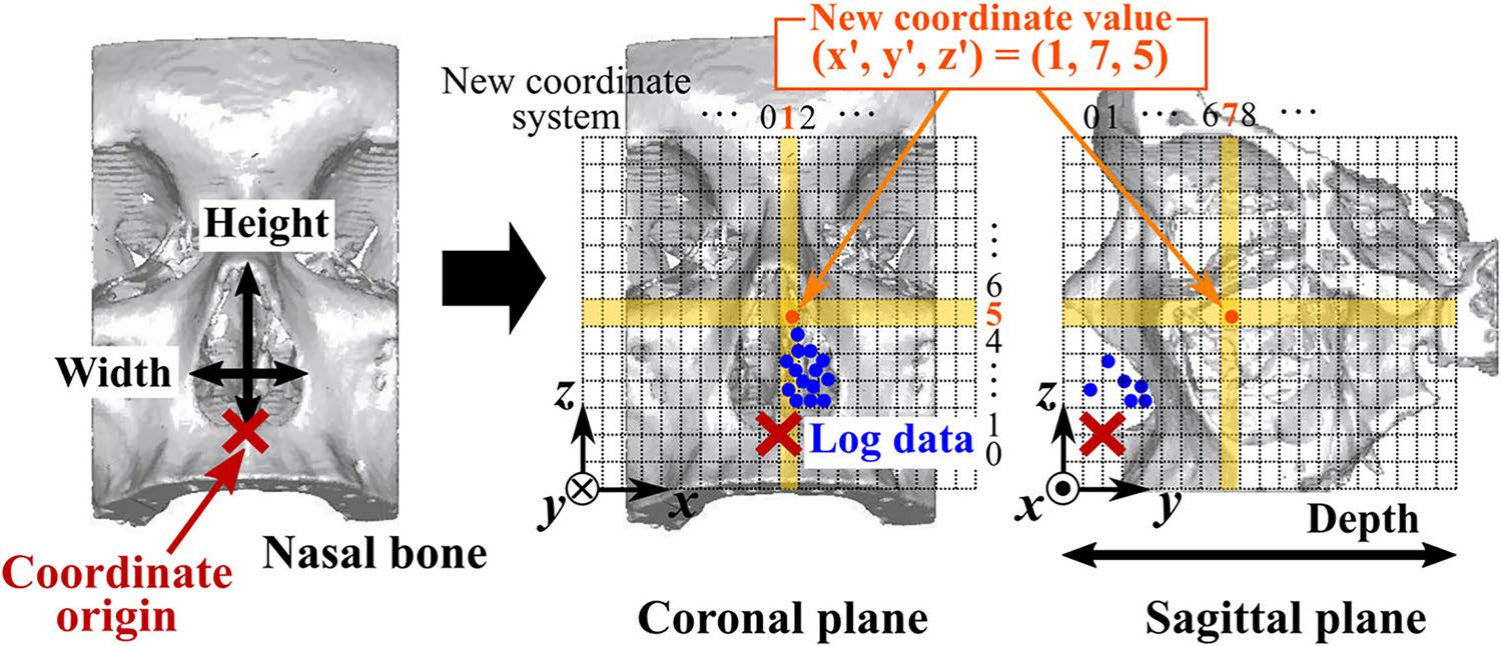
Assignment of the new coordinate values to the surgical log data^7^. Based on the size of the nostrils of the patient, the coordinate system is set so that the number of blocks (i.e., voxels) of the new coordinate system is matched with the nasal cavity in each patient. In the new coordinate system, the coordinates are set for the left and right nasal cavities so that they are symmetrical with respect to the nasal septum. Moreover, the same coordinate value is obtained, regardless of which nasal cavity is treated.

In addition, we used the positional information of surgical instruments. At this juncture, we set a new coordinate system relative to the nasal anatomy, as described in a previous study^6^ (Fig. 2) because the size of the nasal cavities varied among patients. We manually set the origin, axes, and scale of the new coordinate system, based on the position of the anterior nasal spine and the height, width, and depth of the nasal cavity. Then, we used the new coordinate values, $P_{i}^{{\prime }} = \left( { = \left( {x_{i}^{{\prime }} ,y_{i}^{{\prime }} ,z_{i}^{{\prime }} } \right)} \right)$, assigned to the surgical log data as the position information of the surgical instrument. The left and right nasal cavities were assumed to be anatomical symmetric images; thus, the new coordinate values were arranged to achieve symmetry in the nasal septum plane.

The surgical process transition, described as the surgical process model (SPM), is based on the surgical motion and position data. The SPM is defined as follows:

1 $$SPM = \left\langle {ac_{1} ,ac_{2} , \ldots ,ac_{n} } \right\rangle$$

2 $$ac_{i} = \left\{ {\begin{array}{*{20}c} {v_{{R_{i} }} , a_{{R_{i} }} ,j_{{R_{i} }} , r_{{R_{i} }} , x_{{R_{i} }}^{\prime } ,y_{{R_{i} }}^{\prime } ,z_{{R_{i} }}^{\prime } } \\ {v_{{L_{i} }} , a_{L} ,j_{L} , r_{L} , x_{{L_{i} }}^{\prime } ,y_{{L_{i} }}^{\prime } ,z_{{L_{i} }}^{\prime } } \\ {rv_{i} , ra_{i} , rj_{i} , ba_{i} ,bd1_{i} ,bd2_{i} } \\ \end{array} } \right\}$$

We created an SPM for each scene. By using the SPM, we conducted the time-series comparison analysis.

### Alignment and comparison of the SPMs

**Figure 3:**
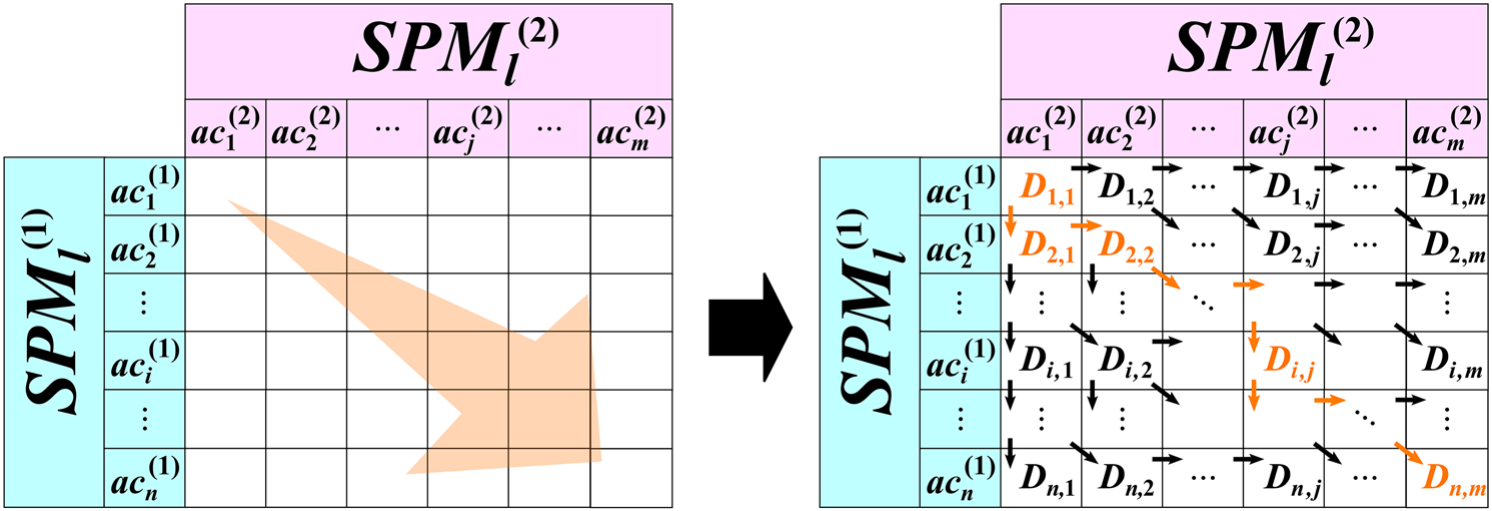
Outline of alignment using DTW. By identifying the optimal path that minimizes the total distance of the Euclidean distance matrix, the two surgical process models can be optimally aligned. *DTW* dynamic time warping.

When comparing the SPMs, the differences in length and order are critical issues. Therefore, we used the dynamic time warping (DTW) method^10^, which can align the lengths of sequences while matching similar parts so that two sequences with different lengths are best matched. When aligning two SPMs [i.e., SPM(1) and SPM(2)], we first calculated the Euclidean distance matrix. The distance is defined as the cost. The cost was also referred to by the similarity and calculated, based on Eq. (3). By determining the optimal path that can minimize the total cost, the two SPMs can be optimally aligned. Figure 3 shows the outline of the alignment, based on the DTW method.

3 $$D_{ij} = d\left( {SPM_{i}^{\left( 1 \right)} ,SPM_{j}^{\left( 2 \right)} } \right) + min\left\{ {\begin{array}{*{20}c} {D_{i - 1} ,D_{j - 1} } \\ {D_{i} , D_{j - 1} } \\ {D_{i - 1} , D_{j} } \\ \end{array} } \right\}$$

In this paper, ${\text{d}}\left( {{\text{SPM}}_{{\text{i}}}^{\left( 1 \right)} ,{\text{SPM}}_{{\text{j}}}^{\left( 2 \right)} } \right){ }$ denotes the difference between the components ${\text{ac}}_{{\text{i}}}^{\left( 1 \right)}$ and ${\text{ac}}_{{\text{j}}}^{\left( 2 \right)}$ of SPM(1) and SPM(2), respectively.

4 $$d\left( {SPM_{i}^{\left( 1 \right)} ,SPM_{j}^{\left( 2 \right)} } \right) = \sqrt {\left( {ac_{i}^{\left( 1 \right)} - ac_{j}^{\left( 2 \right)} } \right)^{2} }$$

**Figure 4:**
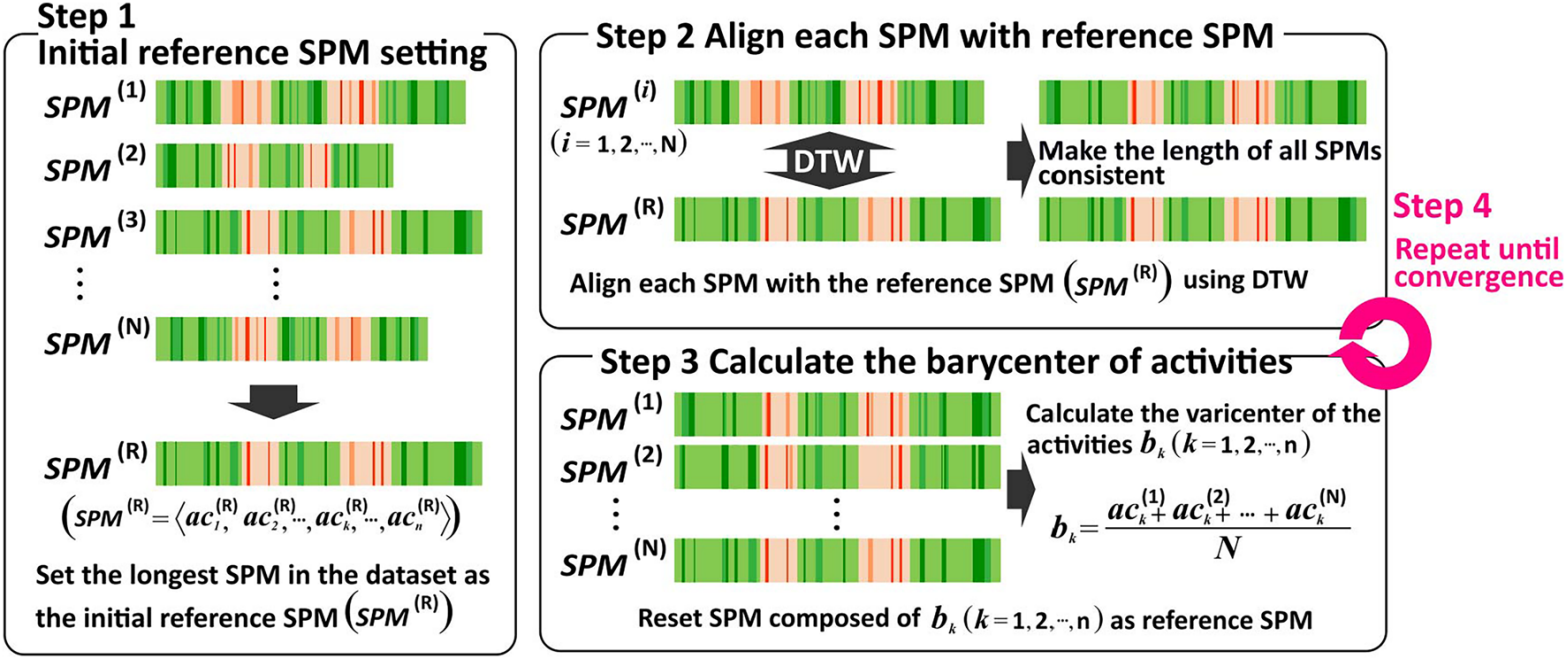
Algorithm of DTW barycenter averaging. Important work may be overlooked if the initial reference SPM is too short. Therefore, we chose the one with the longest operation time for reference. *DTW* dynamic time warping, *SPM* surgical process model.

When comparing the experts and residents, the data on SPM of experts should be standardized because operations performed by experts vary, based on the individual or the surgery. However, the DTW algorithm can only make a one-to-one comparison. Therefore, we used the DTW barycenter averaging (DBA) method^11^, which can be extended to compare three or more sequences, based on the DTW algorithm, and can be used to average the sequences. Figure 4 shows the algorithm of the DBA method. The outline of the DBA algorithm is as follows:

*Step 1* Set the longest SPM in the dataset as the initial reference SPM (SPM(R)).

*Step 2* Align each SPM with the reference SPM and calculate the cost by using the DTW method.

*Step 3* Calculate the barycenter, ${\text{b}}_{{\text{k}}}$, of the activities, ${\text{ac}}_{{\text{k}}}$, included in the SPMs that are aligned to the reference SPM, and reset the SPM composed of ${\text{b}}_{{\text{k}}}$ as the reference SPM.

*Step 4* Repeat Steps 2 and 3 until the alignment costs converge.

Normalization was performed as a preprocess when conducting alignment and comparison of SPMs.

### Experiment

To validate our method, we used the clinical data of 39 patients. We defined an expert as someone who had conducted > 200 ESS procedures. In contrast, a resident had performed < 50 ESS procedures. Nine experts and 19 residents participated.

The average operation time was 12 min 20 s (standard deviation [SD]: ± 5 min 28 s) for an expert and 24 min 3 s (SD: ± 8 min 24 s) for a resident. However, when a patient did not have nasal polyps, Scene 1 was not accounted in the average time calculation.

We created a standard expert model using all data on the experts that were available in the dataset. Next, we compared a single resident SPM with the standard expert model and identified the portions that were significantly different. To avoid detecting instantaneous movements, the detection conditions were empirically set to be an average cost ≥ 8 for 6 s. First, to verify the standard expert process model, we applied the DTW algorithm between the standard process model versus the experts and the standard process model versus the residents. Then, we calculated the average cost between the two SPMs. The closer this cost was to 0, the smaller the difference from the standard process model.

To verify the validity of our methods, the details of the detected parts were confirmed, with the cooperation of expert surgeons, by watching a video of the detected portions. Next, the detected errors were confirmed by putting together videos with similar detection results and showing a representative video. Finally, otolaryngologists reviewed the video to determine any errors other than those detected, as well as any over-detected errors.

### Ethical approval and informed consent

All procedures performed in studies involving human participants were in accordance with the ethical standards of the institutional and/or national research committee and with the 1964 Helsinki Declaration and its later amendments or comparable ethical standards. Ethical approval was obtained from the ethics committee at the Jikei University School of Medicine (Tokyo, Japan) [Approval Number 27-131(8016)]. Informed consent was obtained from all participants included in the study.

## Results

**Figure 5:**
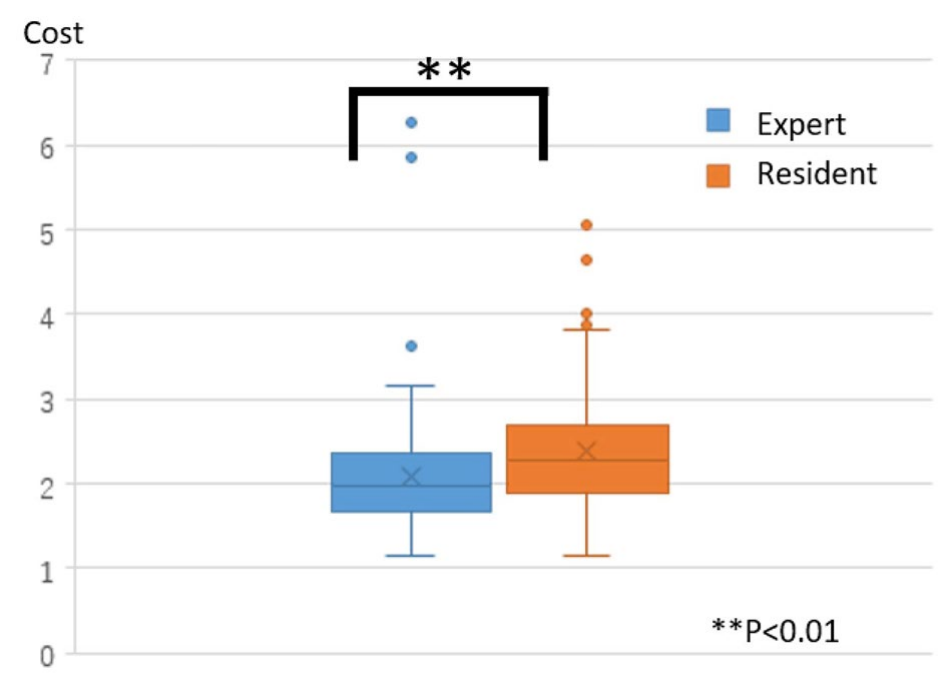
The experts’ and residents’ cost, compared to the standard process model.

After performing the Mann–Whitney U test on the average cost between the experts and the residents (Fig. 5), the cost of surgeries performed by experts was found to be significantly lower than that of the residents. Thus, the difference from the standard process model was small.

After comparing each resident procedure with the standard expert model, 303 portions were detected, with a significant difference that could be improved. After confirming the detected portions using videos, they were classified into the following six categories:

1. Unable to capture the surgical instrument at the center of the endoscope screen.
2. Endoscope and surgical instruments are blurred or interfere with one another.
3. The tips of the endoscope and surgical instrument are very close.
4. Immobility of endoscope or surgical instrument.
5. Variation in treatment position in the corresponding scene.
6. Insertion and evulsion of the endoscope or the surgical instrument.

Excluding the sixth category, the remaining actions were rarely performed by expert surgeons.

Tables 3 and 4 show the details of detected/erroneous portions in the video with significant differences. The average (SD) error time per case was 227.2 (± 137) s. Therefore, the average error rate for each case was 16.1%.

**Table 3:** Details of the detected erroneous parts.

	All	1: Center of the endoscope	2: Blurring or interference	3: Proximity of the tips	4: Immobility	5: Variation in position	6: Insertion and evulsion
Number of detections	303	34	101	57	54	26	31
Rate (%)		11.2	33.3	18.8	17.8	8.6	10.2

The details of the detected parts were confirmed with the cooperation of the expert surgeons.

**Table 4:** Breakdown of errors by scene.

Number of detections/scenes	All	1: Center of the endoscope	2: Blurring or interference	3: Proximity of the tips	4: Immobility	5: Variation in position	6: Insertion and evulsion
1	59	1	16	11	14	14	3
2	40	4	13	8	7	2	6
3	122	17	41	26	17	8	13
4	82	12	31	12	16	2	9

After reviewing the surgery videos, otolaryngologists found that our method missed three errors, which consisted of the following two types: (1) the tip of the forceps was frequently not visible, owing to bleeding and cloudiness of the screen (one place) and (2) the tip of the surgical tool was not visible when performing an excision (two places). In addition, some of the errors detected as “type 5: variation in position” and “type 3: proximity of the tips” may have been overdetected. Thus, a total of 11 overdetections were confirmed. This constituted 3.6% of all errors.

## Discussion

**Figure 6:**
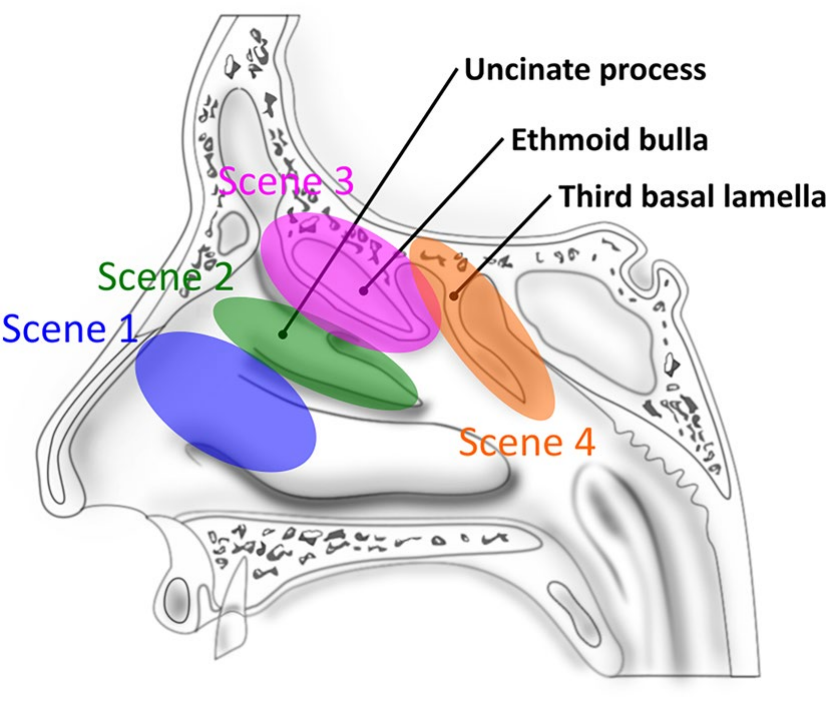
Sagittal view through the ethmoidal sinuses. In endoscopic sinus surgery, the treatment gradually moves to the back of the nose as the operation progresses.

**Figure 7:**
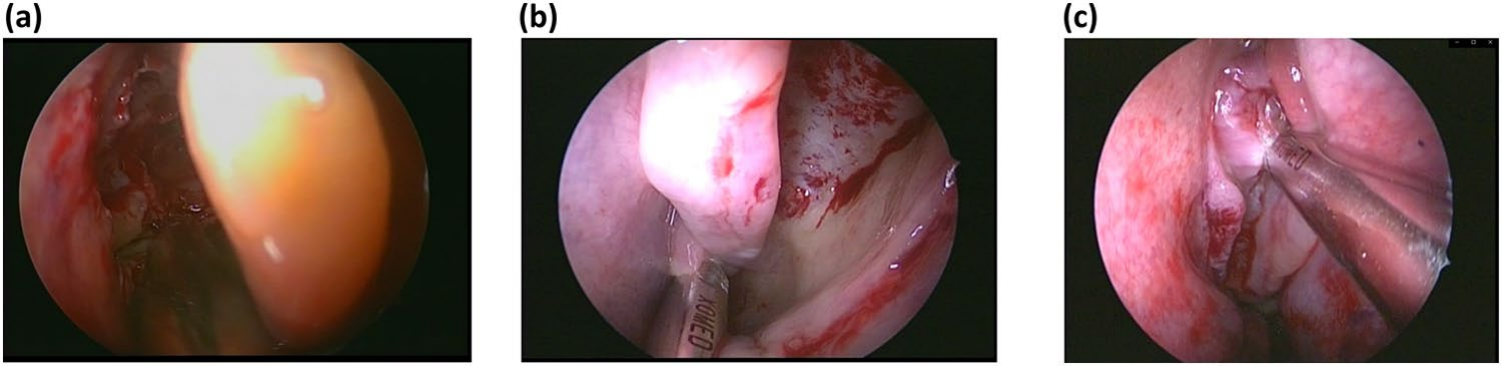
(**a**) and (**b**) Examples of inaccurate treatment positions associated with the different treatment position problem. (**c**) Standard position of an expert, when compared to the position in (**b**). In (**a**), the endoscope is more forward than its intrinsic position. In this situation, the surgical tool damages the middle turbinate. In (**b**), the endoscope is more downward than its intrinsic position, which is shown in (**c**).

The aim of this study was to further develop the time-series comparative analysis method into a multifaceted analysis focused on the motion and position information of the surgical instruments. Furthermore, we aimed to verify the method by increasing the amount of data over the amount used in previous studies^6,7^. As indicated in Fig. 5, the standard process model created during this attempt was close to the expert’s movements, and the details of detected portions were as follows:

1. *Unable to capture the surgical instrument tip at the center of the endoscope screen.* During ESS, a surgeon operates the endoscope with the left hand, and has a surgical instrument, such as a microdebrider or nasal cutting forceps in the right hand. Therefore, surgeons must position themselves in a manner to attain an appropriate endoscopic field of view. However, residents frequently operate without capturing the part to be operated on at the center of the endoscope screen because they are distracted by the actions performed using the right hand. The operation of an endoscope is consequently neglected. When an operation is performed outside the endoscopic field of view, the risk of secondary damage increases. Therefore, the surgical instrument tip must be displayed around the center of the endoscopic screen.
2. *The endoscope and surgical instrument are blurred or interfere with each other.* The surgical tool is generally stabilized by inserting it through the upper nostril, which serves as a fulcrum. During the initial stages of the residency program, the most often cited reason for marginal shifting is interference with the endoscope. However, with practice, the marginal shifting is attributed to the tool being left up in the air because it is not fixed while inserting. When the endoscope and the surgical instrument are blurred or interfere with each other, the endoscopic field of view changes considerably, thereby causing the surgical instrument to move in an unexpected direction and increasing the risk of secondary damage.
3. *The tips of the endoscope and surgical instrument are very close.* Maintaining an appropriate distance between the endoscope and the tip of the surgical tool and operating the endoscope so that the landmarks can be observed are crucial to secure the direction in the nose. However, residents often attempt to enlarge the tip of the surgical tool by bringing it closer to the endoscope. If the endoscope tip and the surgical instrument tip are close, the landmarks in the nasal cavity could be easily lost, thereby obscuring the vertical direction on the endoscopic screen.
4. *Immobility of the endoscope or surgical instrument.* The endoscope or the surgical instrument may be immobile because of various factors, such as when confirming the treatment site or a temporary interruption in the treatment for guidance by the expert. However, experts are familiar with anatomical structures and surgical instruments, thus, exhibit minimal immobility. In addition, the period of immobility decreases with increasing skills.
5. *Variation in treatment position in the corresponding scene.* A previous study^7^ did not detect this type of error, although we speculated that it could have been detected in our study because more parameters were used for analysis. As shown in Fig. 6, during ESS, the part being treated gradually moves to the back of the nose over time. Therefore, certain kinds of problems may occur if the surgery is performed in a position that should not be considered in the ongoing scene. The errors detected during this period could be divided into three types. In the first type, the endoscope is located more forward than its intrinsic position, as shown in Fig. 7a. In this situation, the surgical tool may frequently come into contact with and damage the middle turbinate. Thus, a desirable technique is to hold the middle turbinate with an endoscope and perform the treatment so as to avoid contact. The second type involves incomplete removal of the surgical tool owing to its being stuck in the front of the nose. This complication is rarely observed in surgeries performed by experts who excel in handling these surgical tools. This complication can briefly halt the surgery. The third type of problem occurs when the endoscope is located further downward than its intrinsic position, as shown in Fig. 7b. Experts often perform the procedure with the endoscope at the same height for the right-handed surgical instrument, as shown in Fig. 7c; thus, a possibility is that this difference was detected only in operations performed by residents. After analyzing the videos related to the third problem, experts suggested that improvement was not mandatory for these procedures, although some of these operations were performed in unusual positions. The parts that were significantly different from the standard expert model were detected uniformly; therefore, we believe that we developed an operating model that was considerably different from the procedures followed by experts. However, this does not indicate that improvements will be required in all surgeries performed by residents.
6. *Insertion and evulsion of the endoscope or the surgical instrument.* During surgery, insertion and evulsion of the endoscope and surgical instruments are frequently performed to swap surgical instruments and clean the endoscope. Therefore, in general, whether this part requires improvement cannot be stated. In this study, we set a threshold for the surgical data to exclude the data when the surgical tool was completely outside the nose. However, as the frontal threshold in the depth direction was the apex of the nose, a few extranasal parts were detected in the dataset.

As shown in Table 3, the number of detected parts was high in Scenes 3 and 4. This is thought to be attributed to the fact that as the surgery progresses, the treatment area moves to the back and is closer to important organs, while the range of movement of the surgical instrument becomes narrower, which increases the level of difficulty. In addition, as shown in Table 4, Error #1: “Center of the endoscope” increased as the scene progressed. As the surgery progresses, it approaches important organs, such as the eyes and brain, which is attributed to increased awareness of the right surgical tool. Error #2: “Blurring or interference” also increased as the scene progressed. This is attributed to the fact that the treated area becomes narrower as the surgery progresses, making it easier for interference. Error #4: “Immobility” and Error #5: “Variation in position” were often detected in Scene 1. This is considered to reflect the immaturity of resident's surgical tool operation and planning ability. As it is the first stage of surgery, it may not be possible to plan how to approach it well, and it may take some time to get used to grasping the anatomical structure and handling the surgical tools. Error #3: “Proximity of the tips” and Error #6: “Insertion and evulsion” may occur regardless of the scene. As shown in Table 4, sthese errors occur regardless of the scene. These results are also considered to reflect the immaturity of resident's ability to use surgical tools.

In this study, we added parameters that show the finer part of the movement of the surgical instrument and parameters that grasp the relative distance relationship between the camera and the working position of the surgical instrument as analysis indices. In our results, we detected a higher number of blurring or interference and proximity of the tips than in a previous study^7^. In addition, a new type of error related to the positional relationship of the surgical instrument was detected. Therefore, it is considered that the error related to the movement and positioning of the surgical instrument can be detected in more detail than in the previous research by adding the index.

The findings confirmed that most variations in performance, as described previously, were procedures involving dangers, such as the occurrence of secondary damage. Therefore, the findings suggested that the implementation of our method of automatically improving point detection is advantageous. Many studies have created and used the SPM to predict the surgical process and remaining time in surgery^12,13^; however, not many studies have analyzed the flow of the surgery itself. Forestier et al.^14,15^ created an SPM, in which the data are entered manually using a tablet. In these studies, the experts’ and intermediates’ SPMs obtained from three institutions were clustered for each institution. The overall surgical procedure flow of each institution was analyzed. In addition, they calculated the learning curve by using the time-series changes in the SPM of individual surgeons and evaluated its validity.

In contrast to these previous studies, we created a model of experts, proposed a method of comparing it with residents to automatically find improvement points in the procedure, and showed the possibility of a new use of the SPM. By using this method, a procedure can be improved by determining the variations that can occur for each scene or skill group, and the aspect to be improved can be presented to a surgeon. Thus, we believe that our proposed method for the automatic detection of surgical improvements would quantitatively indicate the surgeon's procedure bottleneck, which may lead to improved surgical procedures. To date, problems in risky areas during surgery have been identified by reviewing a video after surgery. However, this procedure is time-consuming and labor-intensive for a surgeon. Our proposed method can automatically extract problems, thereby reducing the time required to review and improve the surgical technique.

In our proposed method, the SPM was created and compared for surgical operations in ESS. This comparison was possible because all transitions in the surgical procedures were the same (Table 1). Scenes 1–4 are performed as the first step of the operation procedure in ESS, making this approach more versatile in such surgeries. However, when using this model for other surgical procedures, it is necessary to select a procedure that does not show significant changes in the transition of the surgical process and the content of treatment from case to case, or to arrange the transition and content of the treatment to be the same for each case. In addition, we empirically determined the detection conditions; thus, whether the surgical data change must be redetermined. Moreover, the surgical style may differ depending on the expert; therefore, adapting the standard SPM to individual surgical styles will be necessary in the future. Thus, we believe that the proposed method will help automatically detect the location of the improvement more accurately and provide learning support in various surgical procedures, not limited to ESS.

With regard to missed errors, type 1 error may have been missed because only the movement information of the surgical instrument was used as a criterion. More accurate error extraction may be possible after considering the image data related to the surgical scene. Our proposed methods did not use imaging data for the relevant surgical scene. In one part, the error was accordingly overlooked because of the influence. In particular, we overlooked a risky scene wherein substantial bleeding occurred, and the tip of the forceps was not visible for a long time. This error probably occurred because only the information of the surgical instrument was used as the criterion. We believe that imaging data may allow for more accurate error extraction.

In both missed type 2 errors, in the excision surgery, the tip of the surgical instrument was not visible for approximately 1 s. The task of returning to the normal position was confirmed immediately. The error of our method was detected when the average cost for 6 s was above the threshold value of 8; therefore, the momentary error was possibly overlooked.

By using our method, the number of overdetections was greater than that of oversight. If the threshold value and search time are shortened to avoid oversight, the chances of overdetection may increase significantly. Therefore, a trade-off exists between over-detection and oversight. Oversight can lead to serious accidents; therefore, we believe that overdetection is tolerable, compared to oversight.

In conclusion, we developed a standard process model using the SPM and designed a technique for detecting parts with low similarity to the standard process model to improve the procedure. As a result of the verification, this study confirmed that most detections were procedures that could result in dangers such as secondary damage. The proposed method could potentially automatically detect the improvement point location of the procedure and provide feedback to a surgeon. Our future studies will focus on improving the error detection method and building a system that can more clearly present the results to the surgeon.

## Data Availability

The data sets generated during and/or analysed during the current study are not publicly available for privacy reasons, but are available from the corresponding author on reasonable request.
